# Cachexia in Pancreatic Cancer: New Insights to Impact Quality of Life and Survival

**DOI:** 10.3390/nu17193064

**Published:** 2025-09-25

**Authors:** Saunjoo L. Yoon, Oliver Grundmann, Sherise Rogers, Judith M. Schlaeger, Bo Han, Edward Agyare, Diana J. Wilkie

**Affiliations:** 1 Department of Biobehavioral Nursing Science, College of Nursing, University of Florida, Gainesville, FL 32610, USA; diwilkie@ufl.edu; 2 Center for Palliative Care Research and Education (CPCRE), University of Florida, Gainesville, FL 32610, USA; 3 College of Pharmacy, University of Florida, Gainesville, FL 32610, USA; grundman@ufl.edu; 4 Logrreta Cancer Center, Brown University, Providence, RI 02903, USA; srogers102@brownhealth.org; 5 Department of Human Development Nursing Science, College of Nursing, University of Illinois Chicago, Chicago, IL 60612, USA; jschlaeg@uic.edu; 6 Departments of Surgery and Biomedical Engineering, University of Southern California, Los Angeles, CA 90089, USA; bohan@usc.edu; 7 College of Pharmacy and Pharmaceutical Sciences, Florida A&M University, Tallahassee, FL 32301, USA; edward.agyare@famu.edu

**Keywords:** pancreatic ductal adenocarcinoma, nutrition, dietary supplements, cachexia-associated symptoms, malnutrition, biological markers

## Abstract

**Introduction:** Cancer cachexia is associated with systemic inflammation and metabolic derangement, leading to muscle atrophy, which affects over 80% of pancreatic cancer patients, the highest rate among all malignancies, negatively impacting quality of life and significantly reducing survival rate. Malnutrition, skeletal muscle loss (sarcopenia), and imbalanced energy expenditure are indicators of cachexia. No established screening tools in clinical practice are specific and sensitive enough to detect pancreatic cancer in its early stages. **Objective:** This paper aims to provide new insights by examining contributing factors in the development of cachexia and exploring future directions for managing cachexia to improve quality of life and overall survival in patients with pancreatic cancer. **Conclusions:** It is clinically vital to identify nutritional risks and consider aggressive nutritional interventions as soon as pancreatic cancer is diagnosed to (1) stabilize body weight, (2) decrease the disease-associated burden, and (3) improve the quality of life. To support the clinical management of cachexia in this population, more research is needed. Specifically, research is needed to identify biomarkers, such as muscle fiber-related genes, optimize drug delivery tailored to the specific metabolic and molecular profile, combine chemotherapeutic agents with nutritional supplements, and consider non-pharmacological interventions such as acupuncture and exercise specifically for cancer-cachexia patients. A multifaceted approach will help achieve a better quality of life and prolonged overall survival in patients with pancreatic cancer.

## 1. Introduction

Pancreatic cancer, an aggressive malignancy of the gastrointestinal tract, accounts for 3.3% of all cancers and 8.4% of cancer-related deaths in the United States, with a dismal five-year survival rate of 13% in 2025 [1], and it is projected to be the second leading cancer-related cause of death by 2030 [2]. The relative five-year survival rate depends on the stage of pancreatic cancer at diagnosis, where only 3% of pancreatic cancer patients with distant spread of cancer survive, compared to 44% of patients with a localized stage [1]. The most common form of pancreatic cancer is ductal adenocarcinoma (PDAC), which accounts for 85% of all cases, while neuroendocrine tumors (NET) only account for 3–7% [3,4]. NET may present with earlier symptoms if they are functional and impair insulin production, while non-functional tumors usually become symptomatic after substantial growth and metastasize to other tissues [5].

Factors independent of malignant growth that are known to contribute to the progression of cancer, in general, include diabetes, obesity, lifestyle risk factors (smoking and alcohol consumption), genetic predisposition, as well as environmental factors (nitrite-containing meat consumption, occupational exposure to organochlorine or hydrocarbon compounds) [6,7]. Specifically, a long history of diabetes mellitus or a recent onset in older adults, a history of or current smoking, heavy past or present alcohol consumption, obesity, and pancreatitis have been linked to an increased risk for the development of pancreatic cancer [8].

Prognostic factors contributing to disease progression are nutritional impairment [9,10] and unintentional weight loss [11,12], which may advance to cancer cachexia. Malnutrition, skeletal muscle loss (sarcopenia), and imbalanced energy expenditure are indicators of pre-cachexia, which is led by metabolic changes once the patient enters the cachectic state [12].

Cachexia is broadly defined as unintentional weight loss of more than 5% in 6 months, weight loss of more than 2% if the BMI is below 20 kg/m^2^, or weight loss of greater than 2% with concomitant diagnosis of sarcopenia [13]. Cancer cachexia affects over 80% of pancreatic cancer patients, the highest rate among all malignancies [14], and negatively impacts quality of life and reduces the survival rate. The purpose of this article is to summarize the current understanding of cancer cachexia development and the treatments for its prevention in patients with pancreatic cancer to mitigate its impact on quality of life and overall survival.

## 2. Methods

A literature search in PubMed using the search terms “pancreatic cancer” or “pancreatic adenocarcinoma” in combination with “cachexia”, “cancer cachexia”, or “nutrition” identified a total of 2541 articles. Most articles were excluded because they were duplicates, review articles, in a language other than English, or focused on diagnosis and factors contributing to the development of pancreatic cancer. This left 73 original research articles that were included in this review.

## 3. Diagnosis of Pancreatic Cancer and Cancer Cachexia

Early detection of pancreatic cancer is critical in slowing its progression, and early treatment focuses on promoting remission. Although early-stage detection is vital, most pancreatic cancers are asymptomatic until later-stage presentation with non-specific symptoms, including jaundice, weight loss, fatigue, loss of appetite, and abdominal pain [3]. The staging of pancreatic cancer includes tumor localization and size, lymph node involvement, and metastasis formation [7]. Tumors limited to the pancreas and that are either less than 2 cm or equal to or greater than 2 cm with no lymph node involvement or distant metastasis are classified as stage I. Any growth beyond the pancreas (encompassing the duodenum and bile duct, or even the stomach, spleen, and colon) constitutes stages II, III, or IV. Patients who present with significant weight loss tend to be diagnosed at later stages and are more likely to present with cachexia.

Currently, no gold-standard screening tools in clinical practice are specific and sensitive enough to detect pancreatic cancer in its early stages. While only biopsy can confirm the diagnosis of pancreatic cancer, imaging techniques like computer tomography (CT) or magnetic resonance imaging (MRI) as well as serological biomarker Carbohydrated antigen 19-9 (CA19-9 or sialyl Lewis antigen A; normal range 0–37 U/mL) are commonly used to detect a suspected diagnosis of pancreatic cancer and evaluate prognosis and treatment efficacy [15,16]. CA19-9 is primarily limited by a low positive predictive value of 0.9% in the asymptomatic population and its relative non-specificity in distinguishing pancreatic cancer from other malignancies [15]. About 5–10% of patients with a Lewis antigen negative phenotype, indicating no or very low CA 19-9 secretion, will show false negative results [16,17].

**Figure 1:**
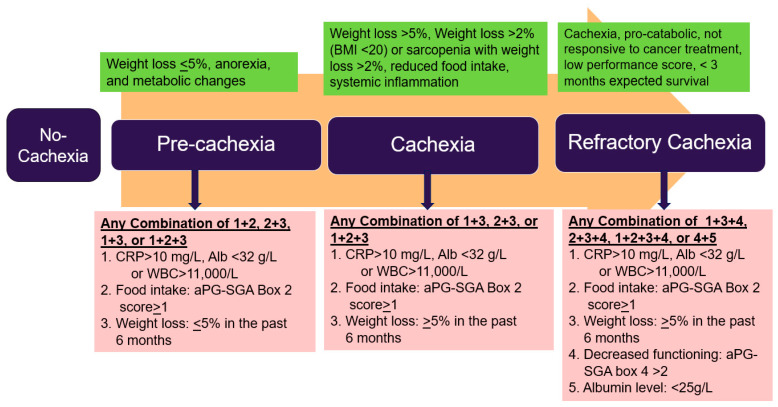
Cachexia stages and the criteria: Criteria 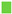 by Fearon et al. [13] and 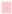 Vigano et al. [19]; CRP: *C*-Reactive Protein, WBC: White Blood Cell Count, aPG-SGA: abridged Patient-Generated Subjective Global Assessment.

Approximately one of every two patients with pancreatic cancer presents with either sarcopenia or pre-cachexia at the time of diagnosis, which requires immediate attention as it impacts the progression of the disease [18]. Cancer cachexia is a multi-symptom complex currently categorized into three stages: pre-cachexia, cachexia, and refractory cachexia (Figure 1) [13].

Pre-cachexia presents with unintentional weight loss less than 5% over 6 months, anorexia, and metabolic changes. The hypercatabolism of pre-cachexia is primarily associated with increased tumor metabolism, systemic inflammation, and pharmacotherapy interventions such as chemotherapy. Pancreatic cancer cells survive in a hypoxic and nutrient-starved environment because they have adapted by inducing autophagy through upregulation of macropinocytosis, a process that involves trapping extracellular nutrients such as lipids and proteins through pinocytosis [20]. This process leads to further starvation of healthy cells, creating a more imbalanced microenvironment that favors the high anabolic activity of pancreatic tumor cells.

Systemic inflammation is generally evaluated using *C*-reactive protein (CRP) and proinflammatory cytokines, including interleukin-6 (IL-6) and tumor necrosis factor α (TNF-α) [21]. Skeletal muscle atrophy is primarily driven by the tumor and its associated microenvironment through multiple signal pathways, including insulin-like growth factor 1 (IGF-1) and transforming growth factor β (TGF-β). These pathways alter catabolic signaling and regulate the interplay between myostatin and activin in muscle cells [22]. The presence of cancer cachexia precludes many patients from a radical resection of the tumor due to pre-surgical sarcopenia and malnutrition and promotes increased post-surgical complications and mortality [14]. It is unclear whether unique pathophysiological changes in pancreatic cancer contribute to the high rate of cachexia or whether a pre-cachectic state is present due to a delayed diagnosis [14].

## 4. Mechanisms of Cachexia and Potential Biomarkers

### 4.1. Mechanisms of Cachexia

**Figure 2:**
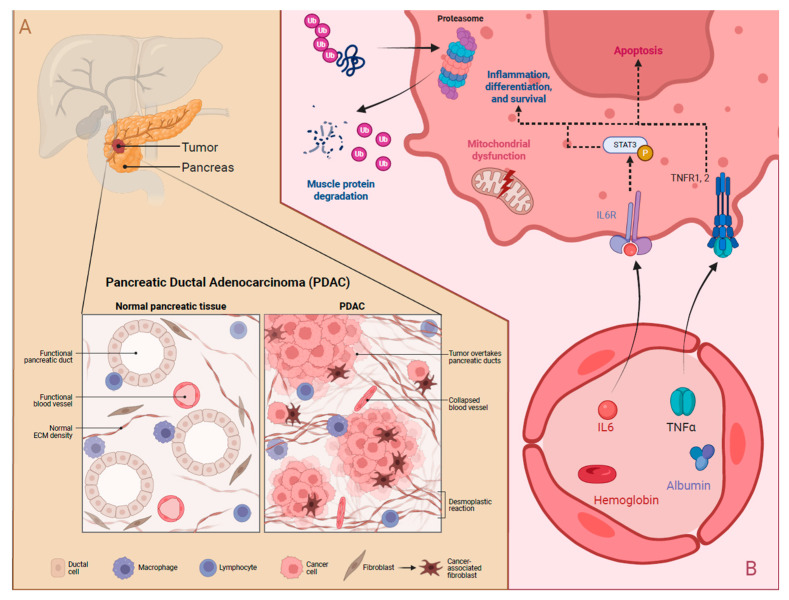
(**A**) Pancreatic ductal adenocarcinoma (PDAC) cancer pathology and (**B**) Pancreatic cancer cachexia mechanisms. ECM: extracellular matrix, IL6: interleukin-6, TNFα: tumor necrosis factor α, TNFR1,2: tumor necrosis factor α receptors 1 & 2, IL6R: interleukin-6 receptor, Ub: ubiquitin.

Cancer cachexia is currently only diagnosed based on skeletal muscle atrophy and weight loss, but it involves complex pathophysiological metabolic changes, including metabolic dysregulation, tumor-microenvironment interactions, and systemic inflammation. The tumor microenvironment and associated increased metabolic demands drive subsequent systemic metabolism and inflammation changes. Figure 2 depicts pancreatic cancer pathophysiology and the simplified cachexia mechanisms.

The tumor microenvironment in PDAC seems to be high in regulatory T and B cells as well as in innate lymphocyte cells, which all create an immunosuppressive environment [23]. Another proinflammatory signaling pathway that appears to be highly prevalent in the pancreatic tumor environment is the transcription factor STAT3, which mediates cell differentiation, proliferation, and apoptosis through IL-6 activation [24,25]. Increased STAT3 expression has been identified in cancer-associated fibroblasts, which are highly prevalent in pancreatic cancer. The importance of the IL-6/STAT3 pathways in pancreatic cancer proliferation has been demonstrated in STAT3 knock-out mice, resulting in significantly reduced development of pancreatic cancer, slower proliferation, and prolonged survival [25]. Although no treatment is currently available to inhibit STAT3 expression or activation, targeting IL-6 has been tested in clinical trials [26]. In a small trial, patients with PDAC stage IV, who received dendritic cells in combination with major histocompatibility complex class I and II peptides in addition to chemotherapy and presented with an immune response, expressed by significantly lower IL-6 levels, had longer overall survival compared to those who did not present with an immune response [26]. Patients treated with gemcitabine, who presented with low IL-6 levels, also had more prolonged overall survival than those with high IL-6 levels. IL-6 levels may serve as an indicator of gemcitabine effectiveness in pancreatic cancer patients [27]. Because of the relationship between systemic inflammatory markers and the tumor microenvironment, the distinction between cachexia and sarcopenia has important implications for disease prognosis and progression. Cachexia is associated with systemic inflammation and metabolic derangement, leading to muscle atrophy, while sarcopenia is solely muscle atrophy without the inflammatory component [28].

A study of 232 patients with advanced pancreatic cancer comparing cachectic patients to non-cachectic patients demonstrated that median overall survival (mOS: 9.430 months, 95% CI: 8.006–10.854) and median progression-free survival (mPFS: 5.950 months, 95% CI: 5.133–6.767) of patients with cachexia were significantly shorter (*p* = 0.005, *p* = 0.036) than those with non-cachexia (mOS: 17.050, 95% CI: 10.504–23.596, mPFS: 8.210, 95% CI: 6.578–9.842), which indicates the negative impact of cachexia [18]. Other contributing factors to decreased survival included hemoglobin levels < 12 g/dL, albumin < 3.5 g/dL, and a BMI < 22 kg/m^2^ [18].

### 4.2. Potential Biomarkers in Pancreatic Cancer Cachexia

Although no biomarkers for the detection of cachexia in pancreatic cancer patients are currently available in clinical practice, early biomarkers to detect pre-cachexia, cachexia, or refractory cachexia have been proposed. One such biomarker is serum paraxanthine, which is significantly lower in pancreatic cancer patients with cachexia compared to non-cachectic patients [29]. To date, this marker has not been evaluated for other cancers, so its sensitivity and specificity remain unclear. However, the aggressive and rapidly progressing nature of pancreatic cancer may account for the discrepancy in paraxanthine levels, which relates to the hypermetabolism of DNA bases. Other general biomarkers include albumin and hemoglobin levels [13], and proinflammatory mediators, especially IL-6 and TNF-α. All of these are not specific to pancreatic cancer, and results may need to be monitored over time in individual patients to predict the development of cachexia.

Emerging evidence indicates that neuromuscular junction (NMJ) degradation, mitochondrial dysfunction, and activation of proteolytic pathways are essential but underexplored contributors to pancreatic cancer cachexia. In a genetically engineered mouse model of pancreatic ductal adenocarcinoma (PDAC), cachexia was associated with the upregulation of denervation-responsive genes, implicating NMJ destabilization and early signs of muscle denervation in the progression of muscle atrophy [30]. Cachectic muscle exhibited pronounced mitochondrial abnormalities, including disrupted cristae architecture and impaired oxidative phosphorylation, suggesting that mitochondrial dysfunction plays a central role in energy imbalance and oxidative stress [31]. Proteolytic systems, particularly the ubiquitin–proteasome and autophagy–lysosome pathways were activated, promoting muscle protein degradation [32]. Together, these findings underscore the importance of neuromuscular integrity and metabolic homeostasis in PDAC-associated muscle wasting, warranting further mechanistic investigation.

## 5. Symptoms Associated with Cachexia Due to Pancreatic Cancer

### 5.1. Symptoms of Cachexia in Patients with Pancreatic Cancer

One of the predominant symptoms of pancreatic cancer, even in its early stages, is involuntary weight loss. In a recent study evaluating the quality of life in pancreatic cancer patients, there were no significant differences in quality-of-life indicators following 9 months of monitoring compared with baseline measures [33]. However, financial difficulties that impact treatment access and are associated with reduced quality of life significantly worsened over time, indicating that patients’ overall physical and psychological well-being decreased. Increased fatigue or gastrointestinal issues in this study were associated with higher mortality and decreased overall survival [33].

At the onset of cachexia in pancreatic cancer, patients experience a range of symptoms associated with the dysregulation of vital pancreatic functions, such as increased gluconeogenesis, decreased pancreatic beta-cell function, pancreatic duct obstruction resulting in abdominal pain, increased lipolysis, altered regulation of appetite, intestinal bacterial overgrowth, and malabsorption of nutrients [34]. Because the initial symptoms are rather non-specific, patients present primarily with fatigue, weight loss, anorexia, jaundice, nausea, and abdominal pain. A formal diagnosis of cancer cachexia is usually one of exclusion. However, several accompanying symptoms complicate treatment approaches, such as a new diagnosis of diabetes mellitus, a family history of pancreatic ductal adenocarcinoma, or chronic pancreatitis, should serve as an indication of further workup to evaluate a patient for pancreatic cancer [35].

Pancreatic exocrine insufficiency, of which diabetes is a symptom, affects more than two-thirds of patients with pancreatic head tumors at diagnosis and increases to over 90% in later stages [34]. The secretion of essential hormones such as insulin and glucagon, which aid in optimal nutrient utilization, is regulated through both parasympathetic and sympathetic innervations. It has been shown that the parasympathetic nervous system increases in insulin release is not impacted as much in pancreatic cancer, and tissues become less sensitive to insulin and glucagon release [36]. The loss in pancreatic hormone sensitivity appears to be directly associated with muscle loss [37].

Malabsorption of nutrients from the intestine is a consequence of insufficient production or expression of pancreatic digestive enzymes. It has been observed in up to 66% of patients with pancreatic cancer at diagnosis [38,39]. Although substitution with pancreatic enzymes and hormones may benefit some patients, it is a short-term and partial treatment to delay the progression of cachexia and sarcopenia [38]. The diagnosis of malabsorption is often associated with weight loss, hypoalbuminemia (serum albumin < 3.5 mg/dL), digestive alterations (constipation or diarrhea, vomiting & nausea, bloating & flatulence), and pancreatic exocrine insufficiency (decreased pancreatic lipase). In addition, patients undergoing pancreaticoduodenectomy or distal pancreatectomy often present with vitamin B12, zinc, and fat-soluble vitamin deficiencies [40].

### 5.2. Current Symptomatic Treatment of Cancer Cachexia

Symptomatic treatment of nausea and vomiting with serotonin type 3 receptor antagonists or atypical antipsychotics can assist in overcoming initial malnutrition [35]. Initial results for using cannabinoids to stimulate appetite have shown promise even in the absence of nausea [41,42]. Cannabinoids may also reduce pain, local and systemic inflammation, and even provide anti-proliferative activity against the tumor itself [43]. There is increasing evidence that the gut microbiome contributes to essential nutrition and immune system processes in both health and disease. Dysbiosis in the oral or gut microbiome may contribute to the development of pancreatic cancer, given the anatomical location of the pancreatic duct where both microbiotas meet [44,45]. Increased abundance of the gut bacteria genus Klebsiella and other Enterobacteriaceae has been associated with an increased risk of pancreatic cancer [45]. Bacterial overgrowth of Klebsiella has been associated with gastrointestinal symptoms and the development of cachexia in a pancreatic cancer mouse model [46]. It may therefore be of use to supplement the diet with specific bacterial strains that can restore the gut microbiome to reduce inflammation and progression of cachexia.

## 6. Impact of Cachexia on Pancreatic Cancer

In a recent study of 309 PDAC cases [47], about 13% were non-cachectic and 87% were in any stage of cachexia (pre-cachexia, cachexia, and refractory cachexia). Patients with cachexia demonstrated shorter overall survival and frequently reported more weight loss, pain, fatigue, anxiety, and depression over time compared to non-cachectic pancreatic cancer patients [47]. The high prevalence of either pre-cachexia or cachexia at diagnosis of pancreatic cancer is because the dysregulation of metabolic processes has often already occurred, and the best approach remains to slow further progression. As a direct consequence of the tumor growth, KRAS-dependent gene mutations in exon 12 profoundly impact metabolic changes in up to 95% of PDAC patients. Consequently, glycolysis and lactate fermentation are increased in the absence of oxygen, favoring the survival and growth of tumor cells [48,49]. This inefficient form of glucose metabolism is known as the Warburg effect and is an essential step in the development and progression of cancer-induced cachexia [48,49]. This tumor-driven effect then leads to catabolic processes in the liver, muscle, and fat tissue that result in cachexia. In contrast, the lipolytic processes in adipose tissues are not understood as well; it remains unclear whether they serve the metabolic demands of the tumor. Small clinical studies indicate that cachectic pancreatic cancer patients present with a reduced ability to oxidize fatty acids as an energy source [50], whereas animal models indicate that increased fatty acid oxidation following a tumor implant may provide energy for tumor growth [49].

## 7. Treatments for Cachexia and Improvement of Quality of Life in Pancreatic Cancer

Pancreatic cancer-associated cachexia is complex, and a multifaceted approach should be used to manage this condition. To date, there are no approved pharmacological therapies or effective medical interventions to treat or reverse cachexia [51], although there is an ongoing effort to develop potential drugs for this condition [52]. Considering the impact of cachexia in patients with pancreatic cancer, it is critical to reduce or slow down its progression by implementing potential interventions if immediate improvement of muscle wasting is not possible. There are various interventions focused on pancreatic cancer patients with cachexia, such as nutritional supplements, pharmacological approaches, and non-pharmacological interventions. These include dietary supplements such as *N*-3-fatty acids, protein supplements, and L-carnitine [53,54,55], parenteral nutritional support [56,57], and pharmacological therapy with thalidomide [58].

### 7.1. Pharmacological Interventions

Thalidomide was investigated in 33 patients with pancreatic cancer using a randomized, placebo-controlled design for 4 and 8 weeks [58]. The intervention group showed positive results in maintaining weight, while the placebo group lost weight. The intervention was more efficacious when used for 4 than 8 weeks, which may indicate a short-term effect.

The treatment options for fatigue are limited in pancreatic cancer patients, although the impact on quality of life is substantial. Mixed findings have been reported with the use of corticosteroids or stimulants like methylphenidate, with transient beneficial effects [59].

### 7.2. Nutritional Interventions

Overall survival based on nutritional risks that were measured with the Geriatric Nutritional Risk Index (GNRI), which used body weight and albumin levels at diagnosis [9,10] and change of nutritional risk over time [9], demonstrated that a group with any nutritional risk showed shorter overall survival than the group with no nutritional risk. The study findings indicated that the increased nutritional risk over time was a predictor of shorter survival in 314 pancreatic cancer patients [9]. An increase in physical function correlated with weight gain (r = 0.56, *p* = 0.001). In one small single-arm interventional study of 32 pancreatic cancer patients [57], parenteral nutritional support (median 18 weeks) resulted in a positive trend of median BMI by 0.8 kg/m^2^, increased phase angle by 10%, or ECM/BCM index (ratio of extracellular mass to body cell mass) with an increase in all three parameters in 9 patients (28%). An unblinded clinical trial with parenteral nutritional support during hospitalization of 100 biliopancreatic mass patients showed weight gain of 1.7 kg (*p* = 0.027) compared to 4.0 kg of weight loss over the previous three months prior to admission [56].

L-Carnitine, converted endogenously to lysine and methionine, has been proposed as a supplement to improve cachexia; in healthy individuals, regular balanced nutrition intake will provide 75% of the required level. In a randomized placebo-controlled clinical trial, L-Carnitine (4 g) intake for 12 weeks in 72 patients with advanced pancreatic cancer increased BMI by 3.4% and improved quality of life compared to the control group (1.5% decrease) (*p* < 0.05) [55].

### 7.3. Non-Pharmacological Interventions

Non-pharmacological interventions for cancer-associated cachexia include exercise [60], acupuncture [61,62], and multimodal interventions such as nutritional therapy combined with exercise [63,64]. A meta-analysis of four exercise intervention studies of 158 cancer patients indicated that strength-based exercise did not significantly increase lean body mass after 8 weeks compared to the usual care groups. Also, the exercise interventions reviewed in the meta-analysis were inconclusive regarding the effectiveness, adherence, and safety for patients with cancer cachexia [60]. In addition, exercise interventions were limited to patients who were functional enough to perform them, and the studies were patient-selective. A randomized controlled study using 30 gastrointestinal (GI) cancer patients with cachexia, including pancreatic cancer, indicated that acupuncture was feasible and may provide weight gain benefits [62]. This finding may have been a potentially gender-specific response due to hormone-specific regulation of food intake [61]. An 8-week acupuncture intervention in 30 patients allocated to receive targeted (focusing on improving cachexia) or non-targeted acupuncture (focusing on using random points not for managing cachexia) was well-accepted with no adverse effects [62]. In a case report, a patient with unresectable body/tail metastatic pancreatic cancer underwent a 12-week multimodal program consisting of exercise, nutrition, and psychological interventions [64]. At the end of the program, substantial improvements in body weight, quality of life, physical fitness, and reduction in distress were reported, while the progression of cachexia was halted. Multimodal approaches to preventing or slowing the progression of cachexia have been shown to impact the overall survival and mortality in patients with pancreatic cancer and should be studied in larger, adequately powered RCTs [65,66].

Overall, most of the non-pharmacologic interventions to manage cancer-associated cachexia showed potential benefits but did not meet the high level of evidence of randomized controlled trials. Most studies were underpowered and provided inconsistent findings regarding weight gain, maintaining muscle mass, or body composition, and did not measure quality of life as a primary outcome in patients with pancreatic cancer. However, the studies indicated that weight stabilization improved survival duration and quality of life in patients with unresectable pancreatic cancer [67] and showed a clinically significant increase in quality of life in patients with GI cancers [68].

## 8. Future Directions and Potential Interventions

Cancer cachexia can lead to alterations in muscle fiber types. Specifically, there is evidence of a shift toward fast-twitch (type II) fibers, which are more prone to atrophy compared to slow-twitch (type I) fibers. This shift likely contributes to the decreased muscle function observed in cachectic patients [69]. Recent research using pancreatic cancer-derived organoids has demonstrated that tumor-secreted paracrine factors can induce dynamic adaptations in muscle fiber type and metabolism, further exacerbating cachexia [70]. For instance, pancreatic adenocarcinoma upregulated factor (PAUF) has been identified as a key mediator of cachexia, promoting muscle degradation in mice through inflammatory signaling pathways [37].

Understanding the mechanisms behind muscle fiber type switching in cancer cachexia is critical for developing targeted therapies to mitigate muscle wasting and improve patient outcomes. For example, zinc supplementation may stabilize cellular membranes and disrupt PAUF’s interaction with Toll-like receptor 4 (TLR4) [71,72], thereby limiting the activation of proinflammatory pathways and reducing the release of cytokines such as TNF-α and IL-6. Additionally, dichloroacetate (DCA), a small molecule with the ability to modulate cellular metabolism, has garnered attention for its potential to shift cells from glycolysis (Warburg effect) back to oxidative phosphorylation, addressing metabolic dysfunction associated with cachexia [73]. Although a definitive biomarker for cachexia remains elusive, in vitro studies have highlighted the potential of using specific muscle fiber-related genes as biomarkers. These biomarkers could guide personalized treatment strategies, helping to optimize therapeutic outcomes for individual patients by tailoring interventions to their specific metabolic and molecular profiles.

In addition to exploring muscle fiber-related genes as biomarkers, innovative treatment approaches are underway to minimize cachexia in pancreatic cancer. Gemcitabine, one of the most widely prescribed chemotherapeutic agents for pancreatic cancer, has been the first-line therapy for almost two decades until recently transitioning into a second-line therapy after the introduction of the combination chemotherapy FOLFIRINOX (folinic acid, fluorouracil, irinotecan, and oxaliplatin) in treating advanced metastatic pancreatic cancer [74,75]. A 2-month study conducted by Cortez et al. showed that a combination of gemcitabine and a ketogenic diet significantly reduced pancreatic cancer-related cachexia in LSL-KrasG12D/+; LSL-Trp53 R172H/+; Pdx1-Cre (KPC) mouse model [76]. This observation highlights the potential of the concomitant use of a dietary regimen and pharmacotherapy in mitigating cachexia in pancreatic cancer.

In a phase II randomized study, pancreatic cancer patients received gemcitabine supplemented with oral eicosapentaenoic acid (EPA), an omega-3 fatty acid. They experienced significantly reduced cachexia and better overall survival in comparison with their counterparts on gemcitabine monotherapy greater than one year [77]. EPA is known to cause the downregulation of proinflammatory cytokines, thereby preventing cachexia [77]. More so, β-hydroxy-β-methylbutyrate (HMB), a metabolite of leucine, as well as glutamine and arginine, may provide attenuation of muscle loss associated with cancer cachexia [78,79]. Dietary supplementation is, therefore, a key addition to pharmacotherapy in preserving muscle mass and improving overall survival in pancreatic cancer.

Cachexia worsens the pancreatic cancer burden and affects patient outcomes. Strategies to optimize drug delivery, such as nanoparticle-based liposomal and polymeric nanoparticle formulations, tumor-targeting capabilities [80], PEGylated gemcitabine, antibody-drug conjugates (ADCs) linking gemcitabine to monoclonal antibodies, and gemcitabine combined with nab-paclitaxel or immune-modulating agents, have demonstrated improved therapeutic response [81], enhanced specificity and reduction of systemic toxicity [82], increased drug retention at tumor sites [83], improved gemcitabine circulation time, and reduced degradation [84]. Studies conducted by Narasimhan and colleagues suggest that Gem/Nab protects against muscle and cardiac wasting in an experimental model of PDAC cachexia [85]. Managing cachexia in pancreatic cancer needs to include nutritional support to improve quality of life and survival [9,78]. Further research is warranted to develop more effective therapies using multifaceted approaches to mitigate the devastating effects of cachexia in pancreatic cancer.

## 9. Conclusions

There has been significant advancement in the reduction of cachexia in pancreatic cancer, which has one of the highest prevalences of cachexia among all cancer types. However, a high prevalence (80%) of cachexia still exists. It is vital to identify nutritional risks and consider aggressive nutritional interventions as soon as pancreatic cancer is diagnosed to (1) stabilize weight, (2) decrease the disease-associated burden, and (3) improve the quality of life. To support the clinical implementation of managing cachexia in patients with pancreatic cancer, further research is warranted to identify biomarkers such as muscle fiber-related genes, optimize drug delivery tailored to the specific metabolic and molecular profile, provide a combination of chemotherapeutic agents with nutritional supplements, and offer non-pharmacological approaches such as acupuncture and exercise interventions. These multiprong efforts will help achieve a better quality of life and prolong overall survival in patients with pancreatic cancer.
